# Threshold Effects of Restraining Factors on China’s Provincial Ecological Footprint in the Process of Urbanization

**DOI:** 10.3390/ijerph17072407

**Published:** 2020-04-02

**Authors:** Decun Wu, Jinping Liu

**Affiliations:** 1 School of Philosophy and Public Administration, Jiangsu Normal University, Xuzhou 221116, China; 2 School of Management, China University of Mining and Technology, Xuzhou 221116, China; jinpingliu@cumt.edu.cn

**Keywords:** ecological footprint, threshold effect, environmental Kuznets curve, urbanization

## Abstract

This study uses a panel threshold model to explore the nonlinear relationship between restraining factors and ecological footprint (EF) evolution from 2003 to 2015 in China. In addition, the heterogeneity of the environmental Kuznets curve (EKC) hypothesis is identified. The results show that the four regime-dependent variables, i.e., technology level, openness, industrial structure and energy efficiency, have significant single-threshold effects on the EF in China, and the negative correlations between these variables and EF are significantly enhanced when the threshold variable urbanization exceeds 86.20%, 68.71%, 86.20% and 47.51%, respectively. As the urbanization level increases, more factors begin to play a high restraining role on the EF. The single-threshold effects on the EKC are significant under the threshold variables of urbanization and industrial structure. Meanwhile, the significant inverted-U relationship trends emerge when the two variables exceed the thresholds of 86.2% and 69.1%, respectively. Based on an empirical study, to restrain the EF of China’s 30 provinces more effectively, the urbanization process should be accelerated, while energy efficiency, foreign capital investment, technology level and service sector proportion should be promoted according to the urbanization level. Compared to other studies, this study is more focused on EF restraining factors and it contributes to the identification of the heterogeneity of EF’s restraining factors and EKC hypothesis, which would be useful for the EF reduction policy in the case of China.

## 1. Introduction

Urbanization initially features an inflow of the population from rural areas and the agglomeration of the population in cities. Furthermore, urbanization is also known as a process of urban changes in the society, economy and environment, and it has important environmental impacts [1]. Urban areas account for less than 3% of land but generate more than 70% of carbon emissions, and global land ecological changes occur in these areas [2]. Broadly speaking, urbanization could be regarded as a comprehensive social process that reflects a multi-dimensional society [3]. Economic research explores the effects of urbanization on economic productivity and industrial structure, while geographic research considers urbanization as a spatial transformation from rural to urban. The social features associated with urbanization include technological progress, lifestyle improvements, economic development and land use change, while the population features are highlighted in the narrow concept of urbanization.

Currently, global urbanization continues, especially in emerging economies such as China, whose proportion of the urban population grew from 36.22% in 2000 to nearly 60% in 2018 [4]. With the rapid development of urbanization in China, more ecological land is occupied and carbon dioxide is emitted, which leads to gradual eco-environmental deterioration. Due to the eco-environmental requirements of the National New-Type Urbanization Plan of China and the country’s responsibility for global emissions reductions, sustainable trends in urbanization should be achieved to restrain the deterioration of the eco-environment in the process of urbanization of China. The relationship between urbanization development and environmental impacts has been widely researched and debated, with some arguing that urbanization is the main cause of eco-environmental deterioration [5]. However, the development of urbanization can also promote the sustainable development of ecological and environmental factors by concentrating the population, inspiring innovation and increasing wealth. Thus, it is necessary to study the relationship between environmental impacts and social and economic factors in the process of urbanization, and measuring the environmental impacts and identifying influencing factors are two key parts.

A considerable amount of sustainability indicators are available in terms of the environment, economy and society [6]. In recent years, most research on the measurement of environmental impacts and their relationships with social factors has focused on the issue of carbon emissions [7,8,9,10]. As global land use change and greenhouse gas effects are global ecological issues and key research issues in the scope of sustainability [2], there are tons of studies from global [11,12] to Chinese-specific [13,14], and only using carbon emissions to measure the environmental impacts of urbanization would lead to an incomplete picture. The ecological footprint (EF) concept was initially proposed by Rees and Wackernagel [5,15] and represents a comprehensive indicator of environmental pressure, and it consists of human appropriation of land, including arable land, forestland, fishing land, grazing land, and built-up land, and the associated carbon emissions [16]. Land ecology and carbon emissions have been combined to perform sustainability evaluations, and EF has been widely used as a sustainability measurement tool [17,18]. In studies on measuring environmental impacts, an increasing number of researchers use EF as an indicator to provide a more comprehensive measurement of environmental impacts and influencing factors [19,20]. There are also some critics for the faculty of EF. The criticisms mainly focus on three aspects, i.e., the indicator accounting itself, the sustainable measurement and its policy value. For the first aspect, many weaknesses for the accounting were pointed out, e.g., the underestimate of bio-capacity in carbon up-taking and the multiple functions of land [21]. For the second aspect, it’s argued that simplified and idealized indicators cannot reflect the sustainable development of the eco-environment, which is a complex issue [22]. The environment indicator is a broader and complex concept and EF can’t offer valid indicators [23], so the EF is not suitable to be used to measure sustainability [24,25]. For policy use, the option stands that EF can’t be useful for policy decision-making. What’s more, there are opinions that EF is totally useless and that it is bad for economics and environmental science [22,26]. Opponents believed that the EF indicator is too simplified and idealized to reflect the actual sustainability, which also would lead to paradoxes in policy decision-making [22]. While supporting viewpoints shows that EF is an objective reflection of the amount of humanity’s ecological use of land, it does not have comparative significance when formulating policies and needs to be used in combination with other indicators, e.g., in the monitoring and early warning of ecology [27]. The GFN (Global Footprint Network) team answered and argued the issues concerning the EF accounting and the role of EF [28,29], meanwhile, improvement and suggestion for EF were proposed and implemented in many studies [21]. Despite much controversy, we think EF would be useful to reflect the pressure on the environment of occupancy to the natural resources. With the pressure of global warming and land use, the local or global eco-environment would benefit from the reduced EF.

Most research has focused on factors that drive EFs, whereas few studies have focused on the factors that restrain EFs. Although exploring the driving forces underlying EFs [30] to achieve the goal of urban environmental sustainability is important, the restraining factors and influencing factors on the formation of a declining turning point must also be identified. To identify this turning point, the environmental Kuznets curve (EKC) hypothesis is widely used to study the relationship between economic development and environmental degradation [31,32]. In general, the EKC hypothesis is tested by judging the coefficient and significance level of the square term of per capita GDP, which can be considered a special influencing factor that is tested if it is a significant restraining factor. Thus, identifying the potential restraining factors of EFs is based on two aspects: the study of social factors’ influences and the study of EKC relationships.

In most cases, improving the technology level, enhancing trade openness [32], increasing the proportion of tertiary industry [33], accelerating urbanization [33], and promoting foreign direct investment (FDI) [34] would be considered as potential restraining factors on EFs. Meanwhile, the inverted-U relationship between economic growth and EFs has been studied. Due to the unbalanced development of urbanization, the economy and society, these aspects will lead to the problem of a lack of treatment for heterogeneity. Due to the heterogeneity of the relationship between EFs and social factors, a number of these factors are used to verify the EKC hypothesis and they are grouped by income level [32] and urbanization rate [31] according to existing grouping criteria. However, the jumping character of the relationship cannot be well captured by existing grouping criteria.

Based on the above, this study tries to identify the heterogeneity and capture the jumping character of the relationship for a study case with individuals of unbalanced development. This study is more focused on EF restraining factors in the process of urbanization compared to others. We adopted a threshold regression model to solve this issue because such models are widely used to capture these types of jumping characters [35]. Due to the unbalanced development of EFs and social factors in China’s 30 provinces, the heterogeneous characteristics of the restraining factors of the provincial EFs of China from 2003 to 2015 under different threshold variables were evaluated. This study has the following objectives: (1) the threshold effect of restraining factors on China’s provincial EFs will be explored under the variable of urbanization; (2) the heterogeneity of the EKC effect will be tested to identify whether there is a threshold effect of social factors on the inverted-U relationship between the EF and economic growth; (3) specific provincial heterogeneity will be analyzed and policy implications will be presented. To achieve these objectives, this article is presented as follows: Section 2 reviews the literature on EKC and EF’s restraining factors. Section 3 introduces the study areas, EF accounting and the threshold panel regression model. Section 4 presents the results of the case study and analyzes the result of the factors affecting China. The final section summarizes the case study, gives policy implications, and provides limitations and directions for future research.

## 2. Literature Review

As discussed in the introduction, most studies on the factors associated with environmental impacts have focused on carbon emissions and economic growth [36], whereas only a few of these studies have used the EF instead of carbon emissions in this scope. As an effective ecological model, EF analysis and accounting are widely used in environmental impact measurement [20,37], sustainability evaluations [38], and policy making and planning [27,39]. Identifying the factors that restrain decreases in the EF is important when the driving factors, such as population growth, cannot be easily reduced in the short term. Thus, we have reviewed literature on the factors that impact EFs and highlighted the restraining (negative) factors for the EF. The literature on the restraining or influencing factors of the EF are listed in Table 1, which shows that most of the studies on the relationship between social factors and EF are analyzed based on the EKC model; moreover, STIRPAT (stochastic impacts by regression on population, affluence, and technology), which was proposed by Dietz and Rosa [40], could be used in conjunction with the EKC for a factor analysis.

Based on the literature listed in Table 1, valid restraining factors for EFs were identified except the square of the economic term. Danish and Wang [20] used the MG-CGE (Mean Group for Common Correlated Effects) for 11 newly industrialized countries and found that economic growth and urbanization had a moderating effect on the EF. The heterogeneity of the social factors that restrain the EF was also explored. Solarin and Al-Mulali [34] explored the effect of the FDI (foreign direct investment) and urbanization on EF evolution for two types of countries and found that FDI and urbanization were restraining factors of the EF for developed countries. Long, Ji and Ulgiati [33] found that the tertiary industry proportion and urbanization were restraining factors for EFs using panel data of 72 countries from 1998 to 2008. Ahmed et al. [41] confirmed the restraining effect of export and foreign direct investment. Similarly, Al-mulali et al. [32] identified financial development, trade openness and urbanization as restraining factors of EFs.

The main idea of the EKC hypothesis is that when the economy develops into a certain high level, environmental pollution tends to be reduced with the increasing economy, namely an inverted-U curve relationship between population and GDP. There are different viewpoints and research paths concerning EKC theory. Regarding the existence of the curve, ecological modernization theory (EMT) believed that with economic and social development, economic development has the ability to overcome ecological and environmental problems, and a curve will emerge. While the pessimistic theory of eco-environment believes that with the development of the social economy, the damage degree of ecological environment will become more serious, and it doesn’t acknowledge the existence of inverted-U curve. Regarding issues of the EKC examining model and empirical study, a lot of deficits were pointed out by Stern [49], e.g., the premise of the hypothesis is too ideal; the model does not consider the feedback of environmental quality on production possibility nor the impact of trade on environmental degradation, therefore the model will underestimate the impact of economic development on the ecological environment [49]. It is argued that most of the empirical study of EKC is weak in econometrics, due to the statistical flaws in the empirical data [49]. The existence of pollution heaven [50] would lead to the formation of the EKC relationship in the high-income countries by transferring embodied pollution to low-income countries. In spite of much controversy on EKC, there are still many meaningful empirical studies and policy implications [36,51].

For the literature related to the EF and the EKC hypothesis, the empirical results could be divided into three types based on the inverted-U relationship. First, the existence of an inverted-U relationship is not supported in a few research studies. Jia et al. [42] used STIRPAT to analyze the factors of Henan’s EF from 1983 to 2006 using the PLS (partial least squares) method to eliminate multicollinearity and they found that an inverted-U relationship did not occur in Henan. Boutaud et al. [43] investigated 131 countries in 2001 using a scatter plot and did not find an inverted-U relationship. Aşıcı and Acar [44] and Bagliani et al. [45] drew similar conclusions in their empirical research. Caviglia-Harris et al. [9] and Aydin et al. [46] also generated similar results in terms of the gross EF; however, they found that the EKC had an effect on components of the EF, with an inverted-U relationship observed between non-energy EF and GDP by Caviglia-Harris et al. and between fishing ground EF and GDP by Aydin et al.

Second, a few researchers support the existence of an inverted-U relationship for all of their study data. Aşıcı and Acar [31] used panel data for 116 countries from 2004 to 2008 to verify the inverted-U relationship between EF and per capita income and a significant EKC was confirmed, and environmental regulation and governance were found to significantly improve the turning point of the EF. Destek and Sarkodie [10] used AMG (augmented mean group) to investigate 11 newly industrialized countries from 1977 to 2013 by separate regressions and they found that accelerating economic growth and urbanization would be helpful for reducing the EF in the study area.

Finally, a few researchers tested the EKC hypothesis by groups according to the income level, urbanization level, etc. to determine the heterogeneity of the existence of the EKC. The inverted-U relationship was supported partially or heterogeneously. Al-mulali et al. [32] divided the major global countries into four categories according to income level and found that the relationship between the square of GDP and ecological footprint varied for different income levels. Specifically, middle- and high-income countries exhibited this type of inverted-U relationship. Ulucak and Bilgili [47] employed the CUP-FM (continuously updated fully modified) and CUP-BC (updated bias corrected) to explore the EKC and confirmed the EKC in all countries for three income levels; however, the EKC turning points were varied.

In addition, Liu et al. [48] used the LMDI (logarithm mean decomposition index) to decompose the factors underlying Beijing’s EF for 2005 and 2010, although they did not identify restraining factors in their study.

Overall, some of the reviewed literature above studied the factors that influence EFs and identified a limited number of restraining factors on EF evolution. A few studies focused on the heterogeneity of the restraining factors and inverted-U relationship based on separate regressions by group. However, most of studies were not dedicated to the restraining factors, and the identification of the heterogeneity in the relationship between the restraining factors and EKC is limited. The essential restraining factors on EFs and associated heterogeneity must be determined to achieve urban sustainable development in a differentiated way during its developing stage.

## 3. Study Areas and Methods

### 3.1. Study Areas

**Figure 1:**
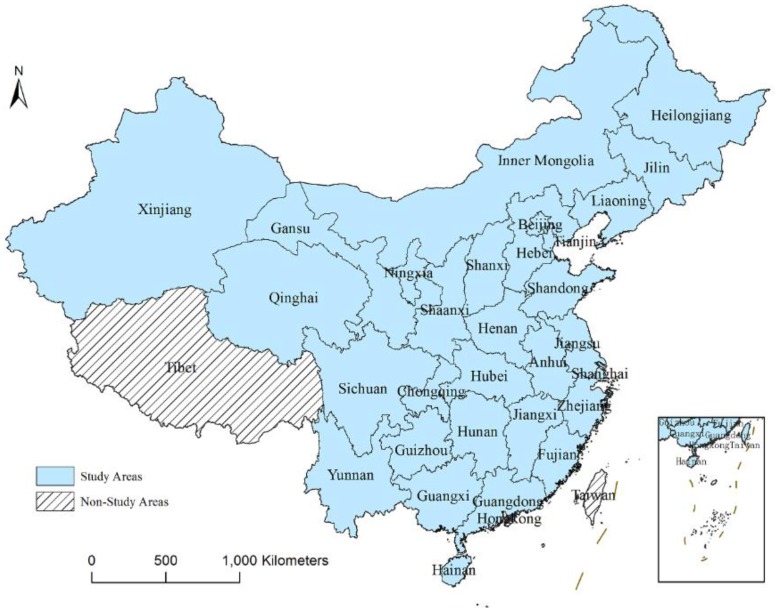
Study areas of China.

In this study, 30 provinces in China (as shown in Figure 1) were selected as the study areas, and due to the issue of data availability, Tibet, Hong Kong, Taiwan and Macao were not included. Ecological data related to the accounting of the EF and social factors in the process of urbanization for the periods 2003 and 2015 in the 30 provinces in China were collected.

### 3.2. Ecological Footprint Accounting

With the macro level of data of Chinese provinces and the available provincial ecological production data, EF accounting of NFA (National Footprint Accounting) [16] was adopted to calculate the provincial EF of China. A provincial overall EF is determined by the sum of the ecological footprint of the imported goods and local goods and the negative value of exported goods. The EF of local products from six types of land is expressed as ${\mathrm{EF}}_{p}$ as follows:

(1) $${\mathrm{EF}}_{P}=\underset{i}{\sum }\frac{P_{i}}{Y_{i}}\cdot {\mathrm{YF}}_{i}\cdot {\mathrm{EQF}}_{i}$$

where $P_{i}$ $Y_{i}\;{\mathrm{YF}}_{i}$ and ${\mathrm{EQF}}_{i}$ represent the total production, local yield, yield factor and equivalence factor for product i, respectively, of cropland, forest land, grazing land, fishing land, and equivalents of built-up land and carbon absorption land. YF represents the annual production per local hector for each product from the cropland, forest land, grazing land, and fishing. EQF is the factor that coverts different land types into a unified unit. The YF and EQF for built-up land and carbon absorption land are the same as cropland and forest land, respectively [16]. As the accounting of the EF is not a key research objective of this paper and has been widely explained, additional details will not be described here and can be found through the GFN [16]. The EFs from imports and exports are calculated in the same way as the EF from local production.

Then, the provincial EF is calculated as follows:

(2) $$\mathrm{EF}={\mathrm{EF}}_{P}+{\mathrm{EF}}_{I}-{\mathrm{EF}}_{E}$$

where ${\mathrm{EF}}_{I}$ and ${\mathrm{EF}}_{E}$ are the EFs from imports and exports, respectively.

### 3.3. Threshold Panel Model

The threshold regression model was proposed and perfected by Hansen [52] using static panel data of 565 companies for 15 years to study whether financial constraints affect investment decisions. Taking a single-threshold model as an example, the double-threshold model and triple-threshold model can be similarly constructed. According to Hansen’s definition, the fixed-effect panel model of the single-threshold regression can be expressed as follows:

(3) $$y_{\mathrm{it}}=\mu +\beta_{1}^{\prime }x_{\mathrm{it}}\cdot I\left(q_{\mathrm{it}}\leq \gamma \right)+\beta_{2}^{\prime }x_{\mathrm{it}}\cdot I(q_{\mathrm{it}}>\gamma )+e_{\mathrm{it}}$$

where $q_{\mathrm{it}}$ is the threshold variable; γ is the threshold parameter to be estimated; I(·) denotes the indicator function; μ and $e_{\mathrm{it}}$ represent the intercept term and disturbance, respectively; and $\beta_{1}^{\prime }$ and $\beta_{2}^{\prime }$ are the coefficients to be estimated. If a significant threshold effect exists, then the relationship of the equation will be divided into two regimes by the threshold variable. Equation (3) can be represented in another form [52]:

(4) $$y_{\mathrm{it}}=\mu +{\beta^{\prime }x}_{\mathrm{it}}\left(\gamma \right)+e_{\mathrm{it}}$$

where $x_{\mathrm{it}}\left(\gamma \right)=\left(\begin{array}{c} x_{\mathrm{it}}\cdot I\left(q_{it}\leq \gamma \right)\\ x_{it}\cdot I(q_{it}>\gamma ) \end{array}\right)$ and $\beta^{\prime }=\left(\beta_{1}^{\prime },\beta_{2}^{\prime }\right)$. To test whether a threshold effect occurs, it is judged that the slope of $\beta_{1}$ and is significantly equal to $\beta_{2}$, and the judging statistical criteria is conducted by F-statistic. The null hypothesis (linear model) is $H_{0}:\beta_{1}=\beta_{2}$, and the alternative hypothesis (single-threshold model) is $H_{1}:\beta_{1}\neq \beta_{2}$.

(5) $$F_{1}=\frac{S_{0}-S_{1}\left(\hat{\gamma }\right)}{{\hat{\sigma }}^{2}}$$

where $S_{0}$ is the sum of squares of the residuals under the null hypothesis; and $S_{1}\left(\hat{\gamma }\right)$ and ${\hat{\sigma }}^{2}$ represent the sum of squares of the residuals and error terms under the alternative hypothesis, respectively. As the threshold parameters are unknown, the bootstrap test proposed by Hansen is performed to calculate the *p*-value by simulating asymptotic distribution for the likelihood ratio test. The bootstrap method is conducted as follows: (i) The residual term ${\hat{e}}_{it}$ is estimated for a given model, then the result of ${\hat{e}}_{it}$ grouped by individuals is used as the empirical distribution of bootstrap sampling; (ii) the distribution is repeatedly sampled as required by the study, then a set of explanatory variable sequences is constructed; (iii) the corresponding F value is calculated for each sample. When all samplings are completed, the *p*-value is calculated by counting the times the F-statistic is greater than the value of $F_{1}$ in Equation (5). When the single-threshold model is not rejected, it is necessary to further test the threshold value. According to Hansen’s method, the likelihood ratio (LR) statistic is used to determine the confidence interval for γ. For ${\mathrm{LR}}_{1}\left(\gamma_{0}\right)$, the null and alternative hypotheses are $H_{0}:\gamma =\gamma_{0}$ and $H_{0}:\gamma \neq \gamma_{0}$, respectively [52].

(6) $${\mathrm{LR}}_{1}\left(\gamma \right)=\frac{S_{1}\left(\gamma \right)-S_{1}\left(\hat{\gamma }\right)}{{\hat{\sigma }}^{2}}$$

At the significance level of α, if ${\mathrm{LR}}_{1}\left(\gamma \right)\leq -2\log\left(1-\sqrt{1-\alpha }\right)$, then the hypothesis of $H_{0}:\gamma =\gamma_{0}$ cannot be rejected. The critical values are 6.53, 7.35 and 10.59 for significance levels of 10%, 5% and 1%, respectively [52].

### 3.4. Design of STIRPAT Regression Model

The STIRPAT [40] model is the stochastic form of IPAT (acronyms of impacts, population, affluence and technology), which was designed to analyze the eco-environment impacts (I) of population (P), affluence (A) and technology (T) in the equation form of I=P·A·T [53]. As an improved and widely used model for factor decomposition of environmental impacts, such as the greenhouse effect and land degradation, STIRPAT overcomes the linear and monotonic nature of IPAT and has been adopted to construct regression models to explore the restraining factors on EFs.

(7) $$I=a\cdot P^{b}\cdot A^{c}\cdot T^{d}\cdot e$$

where a is a constant; b, c and d denote the parameters population, affluence and technology, respectively; and e represents the error term. The equation can also be converted to a linear model in logarithmic form.

(8) lnI=lna+blnP+clnA+dlnT+lne

The term technology (T) is a controversial and indistinct factor compared with P and A [53,54], and it can be used to represent the impact that cannot be explained by population and affluence. Due to the uncertainty of the term T, STIRPAT was constructed or extended in a variety of forms. As the terms P and A are more likely to be driving factors, each potential restraining variable will be constructed in STIRPAT separately as the term T. According to our analysis and the literature review [31,32,33,45,53], the variables urbanization, technology level, energy efficiency, industrial structure and openness of foreign capital were adopted as potential restraining factors in this study, and they are shown in Table 2.

As discussed in the introduction, determining the heterogeneity of the restraining factors is an objective of this study, and panel threshold regression models are designed to test the threshold effect of each restraining factor on the EF. Urbanization’s influence on EF growth tends to be varied among different stages [33,34], and the restraining influence on the EF could come from the effect of resource efficiency and optimization of urban function [33]. To test the influencing effect of different urbanization stages on the EF, we employed urbanization as the threshold variable in the following regression model:

(9) $${\mathrm{lnEF}}_{\mathrm{it}}=\mu +\alpha_{1}{\mathrm{lnP}}_{\mathrm{it}}+\alpha_{2}{\mathrm{lnA}}_{\mathrm{it}}+\alpha_{3}{\left({\mathrm{lnA}}_{\mathrm{it}}\right)}^{2}+\beta_{1}{\mathrm{lnX}}_{\mathrm{it}}\cdot I\left({\mathrm{LnU}}_{\mathrm{it}}\leq \gamma \right)+\beta_{2}{\mathrm{lnX}}_{\mathrm{it}}\cdot I\left({\mathrm{LnU}}_{\mathrm{it}}>\gamma \right)+\varepsilon_{\mathrm{it}}$$

where i and t represent provinces and years, respectively; and X represents technology, openness, industrial structure or energy efficiency. The four threshold regression models were constructed with different values of X. The other symbols can be interpreted based on Table 2.

To study the heterogeneity of the EKC hypothesis between different intervals of variables related to urbanization, four models were constructed to test the threshold effect of the EKC based on the threshold variables of urbanization, openness, industrial structure and energy efficiency.

(10) $${\mathrm{lnEF}}_{\mathrm{it}}=\mu +\alpha_{1}{\mathrm{lnP}}_{\mathrm{it}}+\alpha_{2}{\mathrm{lnA}}_{\mathrm{it}}+\beta_{1}{\left({\mathrm{lnA}}_{\mathrm{it}}\right)}^{2}\cdot I\left({\mathrm{LnY}}_{\mathrm{it}}\leq \gamma \right)+\beta_{2}{\left({\mathrm{lnA}}_{\mathrm{it}}\right)}^{2}\cdot I\left({\mathrm{LnY}}_{\mathrm{it}}>\gamma \right)+\alpha_{3}{\mathrm{lnT}}_{\mathrm{it}}+\varepsilon_{\mathrm{it}}$$

where Y represents urbanization, openness, industrial structure or energy efficiency. With changes of threshold variable Y in equation (10), four models with different threshold variables were constructed.

To process the panel data efficiently and conveniently, the Stata command xthreg was used in the empirical analysis of this study [55]. The double- and triple-threshold regressions model could be constructed similarly.

## 4. Results and Analysis

### 4.1. Results of the Threshold Effect on EF Growth

According to the threshold regression models designed in the previous section, the existence of single-, double- and triple-threshold effects of restraining (regime-dependent) variables were tested under the threshold variable of urbanization. The regime-dependent variables included technology, openness, industrial structure and energy efficiency, which were constructed in model 1, model 2, model 3 and model 4, respectively. These tests aim to determine the heterogeneity of the relationship between EF and each restraining factor among different intervals of urbanization, which were divided based on the threshold regression. According the F-statistics and P-values in Table 3, urbanization had significant single-threshold effects in the four models, and all of the single-threshold tests passed the 5% significant test, indicating the necessity of nonlinear models. For the double and triple thresholds, all four models did not pass the significant test, which indicates that there is only one structural break of urbanization between EF and each restraining variable.

**Figure 2:**
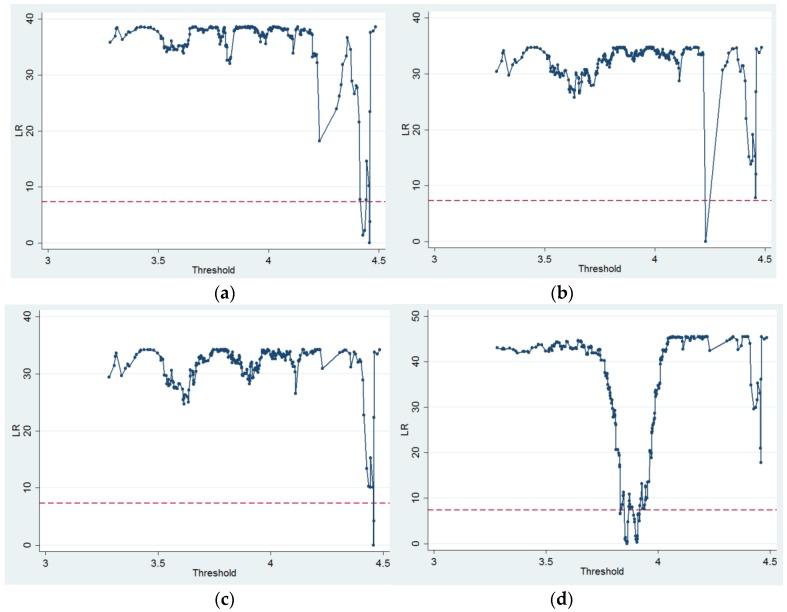
Likelihood ratio of each single threshold of urbanization for each model: (**a**) model 1, (**b**) model 2, (**c**) model 3 and (**d**) model 4.

The likelihood ratio (LR) statistics for single-threshold estimates of urbanization in the four models are presented in Figure 2, and sub-figures (a), (b), (c) and (d) present the LR statistics for single-threshold models from models 1 to 4. The red dashed lines in the sub-figures describe the critical 95% confidence level value. The single-threshold estimates are the value of parameters that obtain a value of zero for the LR statistic [52]. Table 4 shows the threshold estimated value and corresponding urbanization rate for each regime-dependent variable. From model 1 to model 4, the single threshold estimated values of urbanization were 4.4567, 4.2299, 4.4567 and 3.8609, and the corresponding 95% confidence intervals were [4.4415, 4.4578], [4.2195, 4.2966], [4.4539, 4.4578] and [3.8532, 3.8628], respectively.

The results of the threshold regression coefficients from model 1 to model 4 are presented in Table 5. In model 1, the coefficient of technology was −0.0237 in the urbanization rate interval that is no more than 86.2%, which did not pass the significant test. When urbanization exceeded the threshold, the coefficient exhibited a jumping change to −0.1098, which is significant at the confidence level of 1%. The threshold effect of technology indicates that the restraining effect is significant and enhanced only when the urbanization rate exceeds 86.2% for China’s 30 provinces. The jumping change in the coefficients for industrial structure is similar to that for technology. In model 3, the coefficient of industrial structure increased from −0.1 to −0.1588, which represents an increase from not significant to significant at the level of 5%. Model 1 and model 3 shared the same threshold value of urbanization, which indicates the synchronicity on EF reduction for the two restraining factors. The coefficients of foreign capital openness present considerable changes between the two intervals of urbanization in model 2, with the values increasing from −0.061 at a significance level of 1% to −0.3504 at the significance level of 1%. The change indicates that the restraining effect of foreign capital investments becomes much greater when urbanization exceeds the threshold of 68.71%. The situation is similar for energy efficiency in model 4, and the coefficients changed from −0.4762 (lnU ≤ 3.8609) with a significance level of 1% to −0.5788 (lnU > 3.8609) with a significance level of 1%. The restraining effect of energy efficiency was enhanced slightly when the urbanization rate exceeded the threshold 47.51% in model 4. The urbanization factor played a heterogeneous role in the EF reduction effect of each restraining factor. From low levels to high levels of urbanization, the role of technology, openness, industrial structure and energy efficiency in restraining EF growth was enhanced more or less significantly.

### 4.2. Results of the Threshold Effect on EKC

Table 5 shows that the coefficients of the square term of affluence were not significant and were negative simultaneously in the four models, which means that the EKC hypothesis cannot be significantly confirmed for the overall panel data. To further study the specific social conditions for the formation of an EKC between EF growth and economic development, as discussed in Section 3, we constructed four models with different threshold variables based on model 1 to study the heterogeneity in the formation of the EKC hypothesis.

Table 6 shows the single-, double- and triple-threshold effects of the square term under the threshold variables of urbanization, openness, industrial structure and energy efficiency, which correspond to model 5, model 6, model 7 and model 8, respectively. The results show that openness, industrial structure and energy efficiency have significant single-threshold effects at the significance level of 5% according to the F-statistics and P-values in Table 6. Meanwhile, the threshold variable openness passes the 10% significant test. All four models did not pass the significant test for the double- and triple-thresholds tests, which indicates that only one threshold exists. The results denote that the heterogeneity that occured for the EKC and the panel data would be divided into two regimes, between which there are jumping changes for the support of the EKC hypothesis.

**Figure 3:**
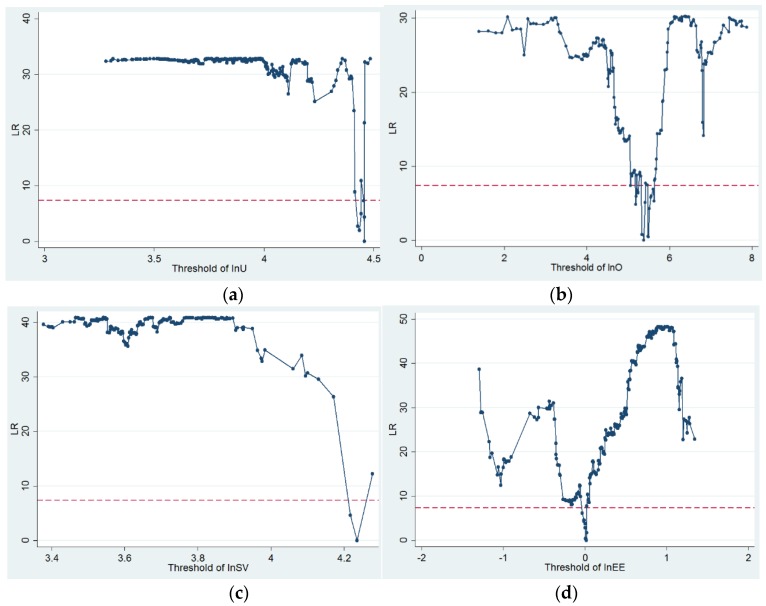
Likelihood ratio of each single threshold for each model: (**a**) model 5, (**b**) model 6, (**c**) model 7 and (**d**) model 8.

Following the test for the presence of a threshold effect, the LR statistic of the four models for single-threshold effect was performed and the LR results are presented in Figure 3. The sub-figures (a), (b), (c) and (d) show the LR statistics for a single threshold from model 5 to model 8. As demonstrated in Figure 2, the red dashed lines in the sub-figures describe the critical 95% confidence level value, and the single-threshold estimates are the values of the parameter that achieved a value of zero for the LR statistic. Table 7 also shows the threshold estimated values and corresponding antilog values for the models. From model 5 to model 8, the single-threshold estimated values were 4.4567, 5.3706, 4.2356 and 0.0164, and the corresponding 95% confidence intervals were [4.4483, 4.4578], [5.3371, 5.4072], [4.1939, 4.2781] and [−0.0231, 0.0175], respectively.

Table 8 presents the threshold regression results on the formation of the EKC relationship between EF and affluence under the threshold variables of urbanization (model 5), openness (model 6), industrial structure (model 7) and energy efficiency (model 8). In model 5, the coefficient of the square term of affluence was −0.0132, which does not pass the significance test when the urbanization rate is no more than the threshold 86.2%. When urbanization exceeded the threshold, the coefficient exhibits a jumping change to −0.0872, which is significant at the confidence level of 1%. The coefficients of the square term in model 6 were 0.0662 and −0.0293 in the intervals before and after reaching the openness threshold of 214.99, respectively. When the per capita foreign capital investment exceeds the threshold for China’s provinces, the formation of the EKC tends to be supported at the significance level of 10%. The jumping change of the inverted-U relationship in model 7 is similar to the situation in model 5. The coefficient of the square term in model 7 changed from -0.021 with no significance to −0.1709 with significance at 1%. The significant turning point of the inverted-U relationship in model 7 is the optimal environmentally friendly evolution. As for the specific heterogeneity of the EKC relationship under the threshold variable energy efficiency in model 8, the coefficients of the square term changed from a positive value (0.131) to a negative value (−0.025) which is not statistically significant, indicating that the EKC relationship is not confirmed. Overall, the growth of the four social factors from low levels to high levels in the process of urbanization plays important roles in the EKC trends for the formation of the EF, and the orders of importance of the EKC formation trends could be industrial structure, urbanization, openness and energy efficiency, successively.

### 4.3. Analysis of the Heterogeneity of Restraining Factors Among China’s 30 Provinces

As shown in the analysis above, the four factors of technology, openness, industrial structure and energy efficiency played a more important role in restraining EF growth when the urbanization exceeded the corresponding threshold. It can be seen that accelerating urbanization promotes the role of these factors, and they each play a specific role. Technology progress is an effective way to reduce the ecological footprint intensity (EFI). Openness improvement brings foreign investment and further brings advanced technology and management, especially for China. Improving industrial structure (service sector’s proportion) is an effective way to restrain EF growth, as the low energy use and land use per GDP for the service sector and the EFI is much lower than that of primary sector or secondary industries. Following the threshold regressions from model 1 to model 4 above, all hypotheses of no single threshold were rejected, indicating significant threshold effects of technology, openness, industrial structure and energy efficiency on EF. China’s 30 provinces could be divided and analyzed to determine the specific heterogeneity based on the specific thresholds. The 30 provinces could be divided into two regimes by each threshold value from model 1 to model 4. The four models share the same threshold variable of urbanization, and the threshold values present a dispersed distribution from low to high. Hence, we divided the urbanization of China’s 30 provinces into four levels based on these threshold values as shown in Table 9: low level, middle level, middle-high level and high level.

The interval of low-level urbanization was no more than 47.51%, and the provinces in the urbanization of this interval in 2015 (latest year in this study) were Guizhou, Gansu, Yunnan, Henan and Xinjiang. None of the factors had a high restraining effect on these provinces in this study. The interval of the middle level urbanization was more than 47.51% and no more than 68.7%, and the provinces that met the criteria included most of the provinces, e.g., Sichuan, Qinghai, Anhui, Hunan, Hebei, Jiangxi, Shaanxi, Shanxi, Hainan, Ningxia, Jilin, Hubei, Shandong, Heilongjiang, Inner Mongolia, Chongqing, Fujian, Zhejiang, Jiangsu, Liaoning and Guangdong. The initial years in which the provinces jumped to the middle level of urbanization varied. While Zhejiang, Jiangsu, Liaoning and Guangdong entered the middle level of urbanization before 2005, Sichuan, Qinghai, Anhui, Hunan, Hebei, Jiangxi and Shaanxi only jumped to this level after 2012. A comparison between the provinces at the middle level of urbanization and those at a low level of urbanization showed that energy efficiency plays a more restraining effect on the EF. Only one province-level city met the middle-high level of urbanization, i.e., Tianjin, and the value ranged from more than 68.71% to no more than 86.2%, and the initial year that this city jumped to the middle-high urbanization level occurred before 2003. With an urbanization rate of 82.64% in 2015, Tianjin will jump to the high level soon. When provinces enter the middle-high level of urbanization, in addition to energy efficiency, the openness begins to play a more important role in restraining EF growth compared with the previous two levels of urbanization which means that the per unit foreign capital investment will have a more restraining effect on the EF. Only Beijing and Shanghai were at the high level of urbanization, which requires a rate of more than 86.2%. As discussed, the same threshold value between technology and industrial structure indicates the close relationships among technology development, the service sector proportion and urbanization, and the two former factors play key roles in the threshold of the high-level urbanization. For the high level of urbanization, in addition to energy efficiency and openness, technology and industrial structure had a high restraining effect on the EF. The per unit technology improvement and increase in the service sector proportion would have a greater restraining effect on the EF in Beijing and Tianjin than other provinces. Due to the unbalanced development of urbanization in China, a heterogamous strategy should be made to restrain EF growth based on the results. For example, the EF restraining effect of technology and openness for the group of Beijing and Shanghai was 4.63 and 5.64 times that of other provinces.

As discussed above, the urbanization rate, openness and industrial structure have a threshold effect on the existence of the EKC for the EF of China’s 30 provinces, and only model 5 and model 7 exhibited a significant inverted−U relationship at the level of 1% when the urbanization and service sector proportion exceeded 86.2% and 69.1%, respectively. Beijing and Tianjin exhibited an inverted-U relationship in consideration of the threshold effect of urbanization, while only Beijing met the service sector proportion criterion of more than 69.1% that supports the EKC with a lower turning point. If the support for the EKC at the significance of 10% in model 6 is considered, then provinces with openness values that exceed the threshold will exhibit an inverted-U relationship to a certain degree, and they include Anhui, Henan, Hebei, Hubei, Sichuan, Chongqing, Jiangxi, Tianjin, Fujian, Shandong, Liaoning, Zhejiang, Beijing, Guangdong, Shanghai and Jiangsu. The EF of Beijing would reach the turning point the earliest, followed by Shanghai, Tianjin and parts of provinces at the middle level of urbanization. The order of provinces is consistent with the urbanization level order from high, middle-high to middle, which indicates that the urbanization rate has a positive effect on the formation tendency of the inverted-U relationship.

## 5. Conclusions and Policy Implications

### 5.1. Conclusions

This paper aimed to study the heterogeneity of the relationship between China’s 30 provincial EFs and associated restraining factors and explore the inverted-U relationship to improve the measures for the sustainable development of urbanization in China. For this purpose, a panel threshold regression and STIRPAT were used to explore the threshold effect on the restraining factors of EFs and the formation of the EKC based on provincial data in China from 2003 and 2015. The main conclusions are as follows:

(1) The threshold effects of the technology level, openness, industrial structure (service sector proportion) and energy efficiency on the EF are all significant and have values of 86.2%, 68.71%, 86.2% and 47.51%, respectively. From a low level to high level across the threshold values, the restraining effects of the four factors were all enhanced, and the jumping character of the restraining effect of openness was the largest. Technology level and industrial structure had the same threshold, which indicates their synchronicity in reaching a high restraining effect on the EF. The distribution of the four thresholds indicates that multistage urbanization has a restraining role on the EF based on different factors. As the urbanization level increases, more social factors have a high restraining effect on the EF.

(2) Urbanization and industrial structure have a statistically significant threshold effect on the formation of the inverted-U relationship between EF and affluence for China’s 30 provinces. The inverted-U turning point will form the earliest when the threshold of industrial structure exceeds 68.71%. The improvement of the urbanization rate not only promotes the formation of the inverted-U relationship but also effectively reduces the EF.

(3) The urbanization of China’s 30 provinces could be divided into four levels, namely, low level (U ≤ 47.51%), middle level (47.51% < U ≤ 68.71%), middle-high level (68.71% < U ≤ 86.20%) and high level (U > 86.20%). Most of the provinces are in the low and middle level, Tianjin is in the middle-high level, and Beijing and Shanghai are in the high level. High restraining effects of all four restraining factors are only observed in Beijing and Shanghai, where a statistically significant inverted−U relationship is supported as well.

(4) The threshold model is an effective way to capture the jumping character of the relationship between EF and its restraining factors. Analysis of the threshold effect is a valid way to study the heterogeneous relationship. This study contributes to the construction of econometric models to identify the heterogeneity of restraining factors and the EKC hypothesis of EF in China. The threshold models could be applied on a global scale or on other pollution indicators.

### 5.2. Policy Implications

Based on the empirical study and the conclusions above, three changes can be implemented to improve the restraining effects on the EF.

(1) The focus on improving technology, receiving foreign capital investments, optimizing the industrial structure and increasing energy efficiency should be phased according to the present urbanization levels of China’s 30 provinces. For provinces at the middle level of urbanization, including Sichuan, Qinghai, Anhui, Hunan, Hebei, Jiangxi, Shaanxi, Shanxi, Hainan, Ningxia, Jilin, Hubei, Shandong, Heilongjiang, Inner Mongolia, Chongqing, Fujian, Zhejiang, Jiangsu, Liaoning and Guangdong, the measures to increase energy efficiency should be a priority. Receiving foreign capital investments should be highlighted for Tianjin at the middle-high level of urbanization. In addition to the two previous points, Beijing and Shanghai should strengthen the restraining effect of technology progress and industrial structure optimization.

(2) For the sustainable development of the inverted-U relationship between EF and economic growth, Shanghai should strengthen the service sector proportion to achieve an optimized EKC tendency. To advance the formation of this tendency, foreign capital investment should be enhanced for Anhui, Henan, Hebei, Hubei, Sichuan, Chongqing, Jiangxi, Tianjin, Fujian, Shandong, Liaoning, Zhejiang, Beijing, Guangdong, Shanghai and Jiangsu.

(3) As an increasing urbanization level would promote the restraining effect of other social factors directly or indirectly, provinces at urbanization levels from low to middle-high should accelerate the urbanization process. Based on the above changes, each province would achieve sustainable development in turn.

## 6. Limitation of This Study and Future Research

### 6.1. Limitations of This Study

There are three limitations of this study: (i) this study uses China’s province-level data to examine the threshold effect of EF’s restraining factors and EKC hypothesis. The sample size is a little small for the study to identify the heterogeneity among regions. It would be more significant and precise to use city-level data on this study. (ii) The ecological footprint indicator mainly focuses on carbon emission and land use, and other aspects of eco-environmental issues were not included in this study, e.g., water pollution and air pollution. (iii) There are some limitations on EKC forms and threshold regression models. For the forms of EKC, this study only considers the square term of the economic term and other forms of EKC were not examined, e.g., EKC model with the cubic term of per capita GDP. Due to a lack of data, policy influences such as environmental protection investment and environmental supervision were not included in this study.

### 6.2. Future Research

Future research could be considered in the following three aspects: (i) multi-scale levels of empirical study could be compared, such as comparing the restraining factors and EKC among nation-level data, provincial-level data and city-level data. (ii) It is recommended to integrate the water footprint and PM2.5 footprint into a more comprehensive footprint indicator for future research, as then the EKC relationship and restraining factors would be identified in a more comprehensive way. (iii) It is worth researching the threshold effect of environmental protection investment and environmental supervision on EF, which would contribute to the direct policy implication.

## Figures and Tables

**Table 1:** Summary of the literature on restraining/influencing factors of ecological footprint.

Authors	Study Area	Data Period	Model	Method	Restraining Factors	Key Findings
Danish and wang (2019) [20]	11 countries	1971–2014	Linear	MG-CGE	Economic growth and urbanization	Economy and urbanization should be accelerated to reduce EF
Solarin and Al-Mulali (2018) [34]	20 countries	1982–2013	STIRPAT	Panel	FDI, Urbanization for developed countries	Effect of foreign direct investment and urbanization on EFs varies between developing and developed countries
Long, Ji and Ulgiati (2017) [33]	72 countries (3 groups by income)	1980–2008	STIRPAT	Static & Dynamic Panel	Tertiary industry proportion, Urbanization	Urbanization brings resource efficiency and environmental awareness
Ahmed, Zafar, Ali and Danish (2020) [41]	G7 countries	1971–2014	Linear	Panel long-run	FDI, Exports	Exports and FDI reduce EFs
Al-mulali, Weng-Wai, Sheau-Ting and Mohammed (2015) [32]	99 countries (4 groups by income)	1980–2008	EKC	Panel Fixed countries and time, GMM	Square of GDP, financial development, trade openness and urbanization	EKC relationship for upper middle-income and high-income
Jia, Deng, Duan and Zhao (2009) [42]	Henan Province, China	1983–2006	STIRPAT, EKC	PLS	None	No EKC exists
Boutaud, Gondran and Brodhag (2006) [43]	131 countries	2001	EKC	Scatter plot	None	Developed countries consume more recourses oversees
Aşıcı and Acar (2018) [44]	87 countries	2004–2010	EKC	Panel	None	No EKC exists
Bagliani, Bravo and Dalmazzone (2008) [45]	141 countries	2001	EKC	OLS, WLS	None	No EKC relationship in quadratic model
Caviglia-Harris, Chambers and Kahn (2009) [9]	146 countries	1961–2000	EKC	Baseline & Dynamic Panel	Square of GDP for non-energy EF	Energy is the main reason for the lack of an EKC
Aydin, Esen and Aydin (2019) [46]	26 EU countries	1990–2013	EKC	PSTR	Square of GDP for fishing EF	No EKC except fishing ground footprint
Aşıcı and Acar (2016) [31]	116 countries	2004–2008	EKC	Panel fixed-effects	Square of per capita income	EKC for per capita income and domestic EFs
Destek and Sarkodie (2019) [10]	11 newly industrialized countries	1977–2013	EKC	AMG	Square of GDP	EKC and bi-directional causality relationship are supported
Ulucak and Bilgili (2018) [47]	45 countries (3 groups by income)	1961–2013	EKC	CUP-FM, CUP-BC	Square of GDP	EKC for countries with low, middle and high income
Liu, Lei, Ge and Yang (2018) [48]	Beijing City, China	2005, 2010	Input-Output for EF Calculation	LMDI	None	Economy, population and footprint intensity are three main driving factors

**Table 2:** List of variables in the regression models.

Variable	Symbol	Explanation	Unit
ecological footprint	*EF*	provincial total ecological footprint	gha
population	*P*	provincial population	10,000
affluence	*A*	per capita GDP of each province, calculated at constant prices in 2003	10,000 yuan
urbanization rate	*U*	proportion of urban population	%
technology level	*T*	authorized patent applications per 10,000 persons	1/10,000 persons
energy efficiency	*EE*	reciprocal of the carbon footprint intensity	10,000 yuan/gha
industrial structure	*SV*	GDP’s proportion of service sector	%
openness	*O*	actual utilization of foreign capital per capita	dollar/person

**Table 3:** Results of the threshold effect test of different regime-dependent variables under urbanization.

Regime-Dependent Variable	Counts of Thresholds	F-Statistic	*p*-Value	Crit10	Crit5	Crit1
*lnT* (model 1)	single	37.79	0.0233	27.3842	31.7908	40.6084
	double	11.41	0.5867	45.4923	60.7859	89.6128
	triple	8.79	0.6800	24.2994	31.1586	59.547
*lnO* (model 2)	single	35.17	0.0300	23.9245	29.1580	40.7600
	double	10.25	0.6800	31.9417	41.5375	64.538
	triple	6.54	0.7933	26.4224	36.1451	48.1326
*lnSV* (model 3)	single	34.71	0.0333	28.8245	32.8041	39.6733
	double	9.04	0.6400	33.8296	50.4759	67.8364
	triple	6.63	0.8000	26.5641	41.9684	76.0287
*lnEE* (model 4)	single	46.16	0.0233	28.5425	36.2342	55.3996
	double	25.56	0.0933	24.5447	32.8072	54.1474
	triple	12.55	0.2500	22.0991	38.9031	55.2277

**Table 4:** Threshold results for each regime-dependent variable.

Regime-Dependent Variable	Counts of Thresholds	Threshold	Lower	Upper	Corresponding Urbanization Rate (%)
*lnT* (model 1)	single	4.4567	4.4415	4.4578	86.20
*lnO* (model 2)	single	4.2299	4.2195	4.2966	68.71
*lnSV* (model 3)	single	4.4567	4.4539	4.4578	86.20
*lnEE* (model 4)	single	3.8609	3.8532	3.8628	47.51

**Table 5:** Test results of the threshold regression models under the threshold variable of urbanization.

Variable	Model 1	Model 2	Model 3	Model 4
*lnT*	−0.0237 (−1.23) (lnU ≤ 4.4567)			
	−0.1098 *** (−4.56) (lnU > 4.4567)			
*lnO*		−0.0621 *** (−2.7) (lnU ≤ 4.2299)		
		−0.3504 *** (−6.94) (lnU > 4.2299)		
*lnSV*			−0.1000 (−1.41) (lnU ≤ 4.4567)	
			−0.1588 ** (−2.21) (lnU > 4.4567)	
*lnEE*				−0.4762 *** (−16.96) (lnU ≤ 3.8609)
				−0.5788 *** (−20.52) (lnU > 3.8609)
*lnP*	1.2952 *** (7.76)	1.8657 *** (9.03)	1.2411 *** (7.50)	0.8169 *** (8.05)
*lnA*	0.5666 *** (13.54)	0.5813 *** (16.78)	0.5270 *** (26.85)	0.5945 *** (42.24)
*(lnA)^2^*	−0.0160 (−0.93)	−0.0239 (−1.4)	−0.0117 (−0.67)	0.0645 *** (5.09)
*C*	7.3474 *** (5.43)	3.1545 * (1.91)	8.1645 *** (6.10)	11.3288 *** (13.76)
*F test*	151.43 ***	136.40 ***	158.39 ***	34.81 ***

***, ** and * are statistically significant at the significance levels of 1%, 5% and 10%, respectively. The values in the first brackets represent the t-statistic. The values in the second brackets represent the interval of the threshold variable.

**Table 6:** Threshold effect test results for each threshold variable.

Threshold Variable	Counts of Thresholds	F-Statistic	*p*-Value	Crit10	Crit5	Crit1
*lnU* (Model 5)	single	33.2	0.0333	26.1934	30.6394	37.5774
	double	12.76	0.5267	56.6116	68.4987	91.5781
	triple	6.52	0.7267	27.2306	35.0821	53.7409
*LnO* (Model 6)	single	30.6	0.1000	30.0675	38.0805	52.1387
	double	14.63	0.4333	24.9354	30.2653	42.1239
	triple	13.19	0.7600	40.0228	51.1239	68.508
*LnSV* (Model 7)	single	41.39	0.0167	25.9956	30.6342	45.1551
	double	6.36	0.5967	37.0055	66.1626	89.715
	triple	5.57	0.6833	30.6904	44.7639	86.3211
*LnEE* (Model 8)	single	48.92	0.0233	34.8738	40.4504	52.3639
	double	23.94	0.1700	27.0381	30.4892	38.8713
	triple	23.58	0.3500	49.2252	62.6302	96.5583

**Table 7:** Threshold results for each threshold variable.

Threshold	Counts of Thresholds	Threshold Values	95% Lower	95% Upper	Corresponding Antilog of the Threshold Values
*lnU*	Single	4.4567	4.4483	4.4578	86.20
*lnO*	Single	5.3706	5.3371	5.4072	214.99
*lnSV*	Single	4.2356	4.1939	4.2781	69.10
*lnEE*	single	0.0164	−0.0231	0.0175	1.02

**Table 8:** Test results of threshold regression on the environmental Kuznets curve (EKC) under each threshold variable.

Variable	Model 5	Model 6	Model 7	Model 8
	Threshold Variable			
	*lnU*	*lnO*	*lnSV*	*lnEE*
*(lnA)^2^*	−0.0132 (−0.77) (lnU ≤ 4.4567)	0.0662 *** (3.05) (lnO ≤ 5.3706)	−0.0201 (−1.18) (lnSV ≤ 4.2356)	0.1310 *** (4.99) (lnEE ≤ 0.0164)
	−0.0872 *** (−3.65) (lnU > 4.4567)	−0.0293 * (−1.67) (lnO > 5.3706)	−0.1709 *** (−5.19) (lnSV > 4.2356)	−0.0250 (−1.48) (lnEE > 0.0164)
*lnP*	1.2718 *** (7.61)	1.0911 *** (7.29)	1.2753 *** (8.02)	0.8719 *** (6.21)
*lnA*	0.5714 *** (13.74)	0.5373 *** (12.67)	0.5727 *** (13.99)	0.5429 *** (13.27)
*lnT*	−0.0269 (−1.4)	−0.0169 (−0.88)	−0.0246 (-1.3)	−0.0088 (−0.46)
*C*	7.5346 *** (5.56)	9.0067 (7.42)	7.5125 *** (5.82)	10.7889 *** (9.47)
*F*	150.61 ***	140.16 ***	150.96 ***	126.57 ***

*** and * are statistically significant at significance levels of 1% and 10%, respectively. The values in the first brackets represent the t-statistic. The values in the second brackets represent the interval of the threshold variable.

**Table 9:** Division of China’s 30 provinces by urbanization level of the threshold effect.

Urbanization Rate (U) Interval	Urbanization Level	Threshed Variable with High Restraining Effects on the EF	Provinces that Meet the Urbanization Criteria in 2015
U ≤ 47.51%	Low	none	Guizhou, Gansu, Yunnan, Henan and Xinjiang
47.51% <U≤ 68.71%	middle	energy efficiency	Sichuan (2015), Qinghai (2013), Anhui (2013), Hunan (2013), Hebei (2013), Jiangxi (2013), Shaanxi (2012), Shanxi (2010), Hainan (2008), Ningxia (2010), Jilin (2005), Hubei (2010), Shandong (2008), Heilongjiang (before 2003), Inner Mongolia (2006), Chongqing (2007), Fujian (2005), Zhejiang (before 2003), Jiangsu (2004), Liaoning (before 2003), Guangdong (before 2003)
68.71% <U≤ 86.20%	middle-high	energy efficiency, openness	Tianjin (before 2003)
U > 86.20%	high	energy efficiency, openness, technology, industrial structure	Beijing (2011) and Shanghai (2005)

The year in brackets indicates the initial year that meets the corresponding urbanization criteria.
